# Predicting clinically significant events in children with ureteropelvic junction obstruction

**DOI:** 10.3389/fped.2024.1409170

**Published:** 2024-05-24

**Authors:** Clara Wolmer, Jean Delmas, Silvia Pecorelli, Eric Dobremez, Cyril Ferdynus, Luke Harper

**Affiliations:** ^1^ Department of Pediatric Surgery, Hopital Pellegrin-Enfants, CHU Bordeaux, France; ^2^ Department of Pediatric Radiology, Hopital Pellegrin-Enfants, CHU Bordeaux, France; ^3^ Methodological Support Unit, Félix Guyon University Hospital Center, Saint-Denis, France; ^4^ INSERM, Clinical Investigation Center-CIC-1401, Bordeaux, France

**Keywords:** hydronephrosis, urinary tract dilation, prognosis, uretero-pelvic junction obstruction, ultrasound

## Abstract

**Introduction:**

Ureteropelvic junction obstruction (UPJO) syndrome is one of the most common causes of neonatal hydronephrosis. Management varies from simple monitoring to surgical intervention, with indications differing between institutions. A consensus of 8 societies recently described a new Urinary Tract Dilation (UTD) classification which aims to standardize ultrasound description of hydronephrosis, but which is also supposed to have predictive value in children with hydronephrosis. Our aim was to compare, in a monocentric prospective cohort of children with UPJO, the ability of UTD to predict the occurrence of a clinically significant event within the first year of life, as compared to anteroposterior diameter of the renal pelvis (APD).

**Study design:**

We used a preexisting cohort of children followed in a prospective study on UPJO. A pediatric radiologist, blinded to the children's outcome, classified the last antenatal ultrasound and postnatal ultrasound according to the UTD-A and UTD-P classification. He also confirmed the APD-A and APD-P measures. We defined a clinically significant event as being: increased pelvic dilation (>5 mm) and/or the presence of a febrile urinary tract infection (fUTI) and/or impaired renal function on initial nuclear scan (<40%). We performed a ROC-AUC curve and Random Forest (RF) analysis to compare the ability of the APD-A, APD-P, UTD-A and UTD-P scores to predict a clinically significant event.

**Results:**

The cohort included 28 children. Clinically significant events were noted in 20 out of 28 patients: 13 children presented an increase >5 mm in dilation, 6 presented an episode of fUTI and 9 had impaired function of the affected kidney. APD-A was the most effective individual criterion for predicting the occurrence of a significant clinical event (AUC = 0.867).

**Conclusion:**

In our series, for children with UPJO, the most significant marker was prenatal APD >15 mm to predict an increase in dilation >5 mm.

## Introduction

Advances in prenatal ultrasound have led to a higher detection rate of prenatal hydronephrosis (1%–5% of pregnancies) (1). Ureteropelvic junction obstruction (UPJO) is the most common cause of hydronephrosis with an incidence of 1/1,500 (2, 3). Typically, UPJO will appear as a dilated pelvis without ureteral dilation, as opposed to obstructive megaureters or high-grade vesico-ureteral reflux which usually present dilated ureters.

Management of children with UPJO is not standardized (4). Surgical indications are fairly unanimous in symptomatic children (febrile urinary tract infections, progressive loss of function or pain), but the management of asymptomatic children is more controversial (2, 5). Some authors will advocate for preventive surgery to avoid future symptoms or loss of function but this does entail a high variability in reported indications and guidelines (6). The management of children with UPJO would be clearer if there were reliable predictive factors of significant future clinical events. Ideally, these predictive factors would be gathered form the prenatal or early postnatal ultrasound data as this is the standard test all these children undergo.

In 2014, following a consensus meeting including American College of Radiology, the American Institute of Ultrasound in Medicine, the American Society of Pediatric Nephrology, the Society for Fetal Urology, the Society for Maternal-Fetal Medicine, the Society for Pediatric Urology, the Society for Pediatric Radiology and the Society of Radiologists in Ultrasounds, a new classification of antenatal hydronephrosis was proposed: the Urinary Tract Dilation (UTD) (7). It was devised originally to help standardize the ultrasound description of urinary tract dilations, but has also been used as a predictive tool, though mainly in rather heterogeneous patient populations and with heterogeneous outcomes.

The aim of this study was to assess the ability of the UTD score to predict the occurrence within the first year of life, of a significant clinical outcome (defined as: significant increase in dilation >5 mm, presence of a febrile urinary tract infection, or presence of impaired function on renal scan) in a homogeneous population of patients with UPJO, and compare it to the “classic” anteroposterior diameter of the pelvis (APD).

## Material and methods

### Patients

Our study population is a population of children, from our institution, currently participating in an ongoing multicentric prospective trial. These are all children who were born between January 2018 and February 2022 and have a standardized follow-up.

Inclusion criteria were: patients presenting with unilateral pelvic dilation, without ureteral dilation, detected prenatally and confirmed postnatally. Prenatal dilation was defined by a renal pelvis measured at more than 4 mm in the 2nd trimester of pregnancy [between 16 and 27 weeks of gestational age (GA)] and at more than 7 mm in the 3rd trimester (from 28 weeks of GA) (6–9). Postnatally, dilation had to be ≥15 mm with a thin ureter (<4 mm) on an ultrasound scan performed during the first month of life for the child to be included in the protocol.

### Follow-up

These patients subsequently underwent baseline ultrasonography at our center and MAG-3 (Mercaptuacetyltriglycine) renal scans between the 4th and 8th weeks of life.

Follow-up was standardized during the first year of life, with consultation and renal ultrasound at 3, 6 and 12 months at our center. No additional nuclear scans were performed during this period. All children continued to be followed every 6 months with an ultrasound after the first year of life.

All patients were initially managed conservatively, and surgery could be performed only if the children presented a significant increase in dilation >5 mm on follow-up ultrasounds or a febrile urinary tract infection (fUTI). None of our patients underwent a voiding cystogram.

### Methods

A pediatric radiologist, blinded to the patient outcome, performed a centralized review of the last preterm ultrasound as well as the 1-month postnatal ultrasound (which was always performed in our institution). He determined the antenatal and postnatal diameter of the pelvis (APD-A and APD-P) and the UTD classification (UTD-A and UTD-P). We did not use the first postnatal ultrasound as it was often performed outside our center and was not standardized.

The APD score was determined by measuring the largest anteroposterior diameter of the pelvis within the hilum on transverse sections of the kidney. The UTD score was measured according to the recommendations of the reference article (7).

### Main outcome

The primary outcome was the ability of the APD-A and -P and UTD-A and -P scores to predict, during the first year of life, the occurrence of a clinically significant event, defined as: a significant increase in the anteroposterior diameter of the pelvis (>5 mm), and/or a fUTI and/or asymmetry of renal function on initial nuclear scan (defined as function of the affected kidney <40%). These three criteria were chosen because on one hand they can constitute for some teams an indication for surgery and on the other because they are more objective than simple “need” for surgery.

Increased dilation was judged on follow-up ultrasound scans and in relation to the reference ultrasound scan performed in our institution. APD measures were defined as the largest diameter at the boarder of the renal hilum in the transverse plane. fUTI had to be documented by fever (temperature >38.5 °C), an increase in C-reactive protein (CRP) >4 mg/L and a positive urinalysis (performed by catheterization, with leukocyturia >10,000 leukocytes/ml, monomicrobial culture and bacteriuria >1,000 colony-forming units/ml). Impaired renal function was determined on initial MAG3 renal scans. There were no repeat MAG3 renal scans meaning we identified impaired function and not decrease in function.

### Statistical analysis

Qualitative variables were expressed in terms of numbers and percentages. Quantitative variables were expressed in terms of mean and standard deviation from the mean, or median and interquartile range. Bivariate comparisons of categorical variables were performed using Pearson's Chi-square test or Fisher's exact test, depending on the conditions of application.

Bivariate comparisons of quantitative variables were carried out using the Mann–Whitney test or Student's *t*-test, depending on application conditions.

The predictive capacity of the different markers (APD-A, UTD-A, APD-P, UTD-P) was estimated, for each judgment criterion, using 1-a logistic regression model and 2-a machine learning model (random forests). Comparisons of predictive ability were made using areas under the ROC (Receiver Operating Characteristic) curve. Comparison between curves was performed according to the method described by Zou et al. (10).

All hypotheses were tested two-sided at the threshold of 5%. Analysis was performed using SAS 9.4 software (SAS Institute Inc).

The study has been approved by the ethics committee.

## Results

The series included 35 children: 7 were excluded for lack of available antenatal ultrasound images. 28 children were included (22 boys and 6 girls) (Table 1). There were 16 patients with UPJO on the left side and 12 on the right. None of the boys were circumcised. None of the patients were lost to follow-up.

**Table 1:** Population data.

Patient characteristics	
Number	28
Boys	22
Girls	6
Side of UJPO	
Left	16
Right	12
Antenatal ultrasound scans	
Date (mean in weeks of GA)	33 weeks of GA
APD-A (mean in mm)	18 mm (9 mm–37 mm)
UTD-A	
UTD-A1	2
UTD-A2-3	26
Postnatal ultrasound scans	
Date (mean in weeks of life)	6 weeks
APD-P (mean in mm)	20 mm (3 mm–55 mm)
UTD-P	
UTD-P1	1
UTD-P2	8
UTD-P3	19

Antenatal ultrasound was performed at an average of 33 weeks of GA, with the majority of ultrasounds performed in the 3rd trimester and only 2 ultrasounds performed at the end of the 2nd trimester. The reference postnatal ultrasounds were all performed between 4 and 8 weeks, with an average of 6 weeks.

The average APD-A was measured at 18 mm (9 mm–37 mm). Ten out of the 28 patients had an APD-A <15 mm and 12/28 had an APD-P >20 mm. Two children were classified as UTD-A1 and 26 as UTD-A2-3. The mean APD-P score was 20 mm (3 mm–55 mm). There was 1 child with UTD-P1, 8 with UTD-P2 and 19 with UTD-P3.

It should be noted that 6 children who had dilations ≥15 mm on the first postnatal ultrasound, which justified their inclusion in the study, had dilation <15 mm on the reference ultrasound performed in our institution. All children had delayed emptying on nuclear scan (residual activity at 20 min >50% of maximum). None of these children were excluded.

Clinically significant events were noted in 20 out of 28 patients (Table 2): 13 children presented an increase >5 mm in dilation, 6 presented an episode of fUTI and 9 had impaired function of the affected kidney. Out of these 20 children, 8 children combined 2 events: 6 had increased dilation associated with impaired function of the affected kidney. One child had increased dilation with fUTI. One child had impaired renal function with a fUTI.

**Table 2:** Clinical events by scores.

	All	APD-A <15 mm	APD-A ≥15 mm	APD-P <20 mm	APD-P ≥20 mm
At least one event		4/10 (40%)	16/18 (89%)	10/16 (63%)	10/12 (83%)
Increased dilation >5 mm	13	0/10	13/18	5/16	8/12
fUTI	6	4/10	2/18	5/16	1/12
Impaired renal function (<40%)	9	1/10	8/18	3/16	6/12

	All	UTD-A1	UTD-A-2-3	UTD-P1	UTD-P2	UTD-P3
At least one event		1/2 (50%)	19/26 (73%)	0/1	5/8 (63%)	15/19 (79%)
Increased dilation >5 mm	13	0/2	13/26	0/1	3/8	10/19
fUTI	6	1/2	5/26	0/1	2/8	4/19
Impaired renal function (<40%)	9	0/2	9/26	0/1	2/8	7/19

Increases in dilation were identified on ultrasound at 3 months for 7 children, at 6 months for 2 and at 12 months for 4 of the children (mean 6 months). The average increase in dilation was 10 mm ± 5 mm. fUTI occurred at 5, 6, 8 and 10 months respectively for 4 children and at 12 months for the other 2 children (mean 8.8 months). All infections were caused by Escherichia coli except one which was caused by proteus mirabilis. Children who presented fUTI or significant increase in dilation underwent surgery. Outcome was favorable for all patients, with no need for further surgery. Interestingly, none of the other conservatively managed patients presented any fUTI or increase in dilation during their second year.

**Figure 1:**
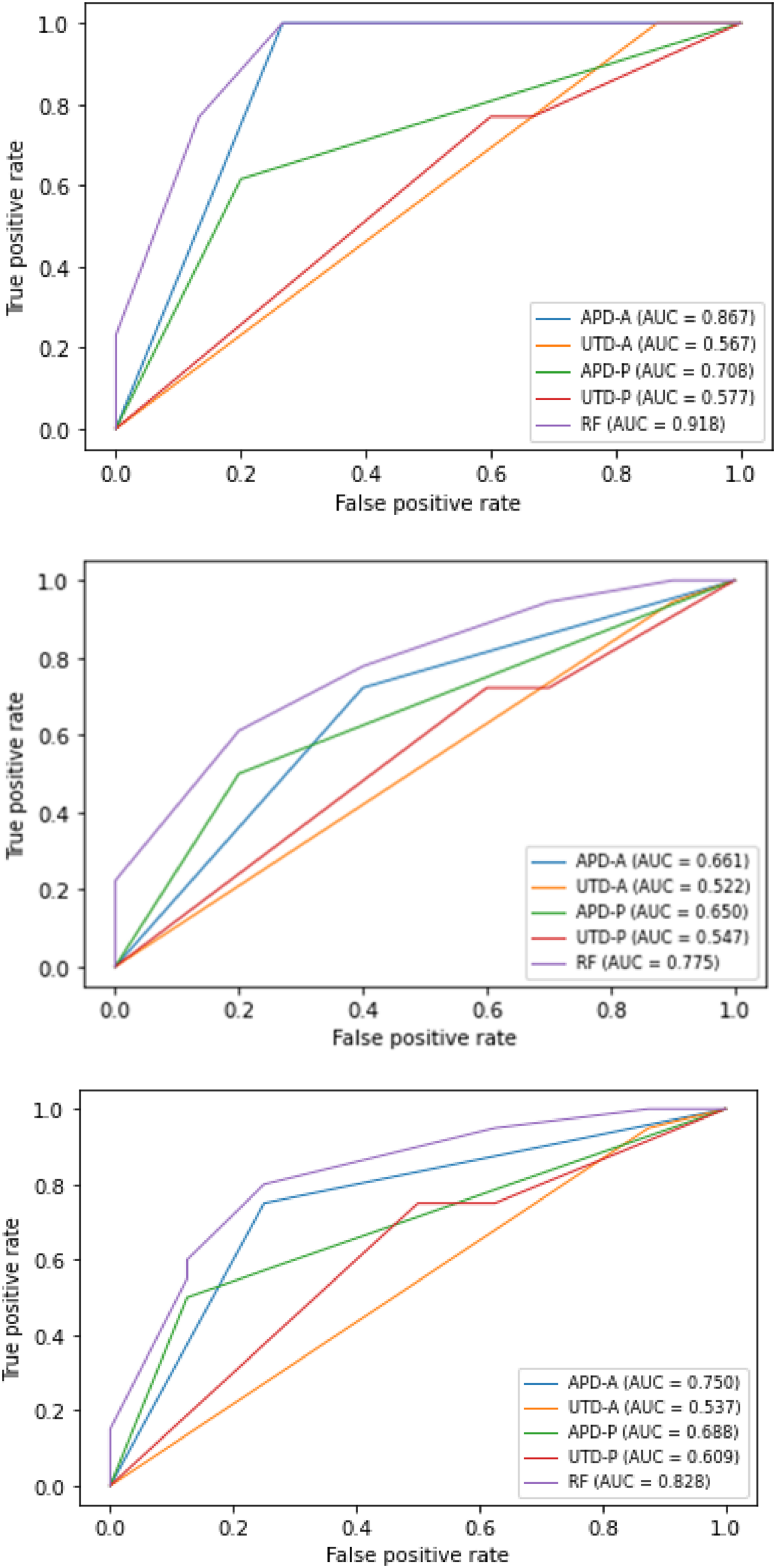
ROC curves representing the predictive ability of the different markers APD-A and -P, UTD-A and -P as well as the four markers together (RF) to predict the occurrence during the first year of life (from top to bottom): (1) increase in dilation by more than 5 mm (2) increase in dilation by more than 5 mm and/or febrile urinary tract infection (3) increase in dilation by more than 5 mm and/or febrile urinary tract infection and/or functional asymmetry (kidney function achieved <40% on scintigraphy).

Analysis of the predictive capabilities of each of the markers studied is illustrated in the three figures below (Figure 1). The thresholds chosen were 15 mm for APD-A and 20 mm for APD-P. On the ROC curves, APD-A was the best predictor of patient outcome, followed by APD-P, then UTD-P and -A. The most predictable event was an increase in diameter >5 mm with an AUC of 0.87 for APD-A (*p* < 0.05). Combining the 4 markers in the “random forest (RF)” increases the predictive score up to 0.92, but mainly under the effect of APD-A.

## Discussion

The scores obtained in our study range from 0.52 to 0.92. A predictive score of 0.8 or above can be considered good. This score was obtained only by APD-A to predict an increase in renal dilation >5 mm. We also obtained a score >0.8 using RF though this was mainly the effect of the APD-A.

In our study, the UTD score did not provide any additional benefit compared with the anteroposterior diameter of the renal pelvis. This is comparable with other studies directly comparing APD and UTD (11). The UTD score was created with the aim of developing a classification system for prenatal and postnatal dilations of the urinary tract, with a reproducible standard terminology, but it has recently been suggested that it be used as a predictive tool for postnatal outcome (7). In 2021, the authors of the original paper concluded that “7 years after its implementation, research has shown that the UTD classification can predict clinical outcomes and that its inter- and intra-rater reliability is similar to or better than those of other systems” (12). Their conclusions were based on several studies that assessed the correlation between UTD classification and clinical outcome. These include studies by Bratina and Kersnik Levart (13), Zhang et al. (14) and Kaspar et al. (15), and especially the study published in 2019 by Nelson et al. (16). In the latter, they investigated the ability of the UTD score to predict the outcome of 494 children (aged 0–90 days) who underwent an initial ultrasound evaluation for antenatal diagnostic urinary tract dilation. Outcome was composite and included the occurrence, during follow-up, of any of the following clinical or diagnostic events: presence of fUTI, vesicoureteral reflux (VUR), UPJO, obstructive mega-ureter, ureterocele, subvesical obstruction and/or chronic kidney disease. The authors observed a correlation between UTD grades and their outcome, as well as a correlation between UTD grade and likelihood of surgical intervention, or UTD score and 3-year resolution of dilation. However, the study was carried out on a highly heterogeneous population. Indeed, among the patients, 1/4 had a normal postnatal ultrasound, 1/4 had an ultrasound with pelvic dilation between 10 and 15 mm with a total of only 6% who were labelled as having UPJO. The studied outcomes were also very heterogeneous, as they included characteristics such as presence of reflux on cystography or a ureterocele, which are diagnostic but not prognostic. In fact the likelihood of surgical intervention for normal and P1 UTD was 1% and it has been suggested that normal and P1 should be combined. A recent review and meta-analysis on UTD by Hae Won Kim et al. on Urinary tract Dilation system noted that higher urinary tract dilation grade was associated with surgical intervention and UTI. However, indications for surgery, were not standardized, and definition of UTI was unclear. Furthermore though the UTD is a four-grade classification, it was essentially being analyzed as a two-grade classification by grouping P0 with P1 and P2 with P3 (17).

It is not surprising that the APD score alone is more relevant than the UTD for UPJO syndromes, as the ureter is not dilated in this pathology and the bladder is normal. In other uropathies, such as obstructive mega-ureters or vesical-ureteral reflux (VUR), the UTD score is likely to outperform the APD score as it identifies ureteral dilation, the degree of which reflects the severity of the condition. In this respect, UTD should probably not be used as a disease-specific tool. It is probably most useful for primary care providers to determine, in the general population of children with ultrasound abnormalities, which are those that require closer follow-up or further investigations and referral. In a recent study of the management of “hydronephrosis” by pediatric urologists worldwide, respondents expressed a clear preference, with regional variations, for the APD or Society of Fetal Urology (SFU) score vs. the UTD score. The authors of this study also noted an impact of the degree of communication between urologists and radiologists and the score used. This degree of communication was rated as high in Europe, where the score most commonly used is the APD (18).

Furthermore, a study published in 2010 showed different thresholds for both diagnostic and therapeutic management between Europeans and Americans, with a tendency for Europeans to wait for higher dilations to investigate or treat prenatally diagnosed renal dilations (19). The specialty of the practitioners caring for these children also has a significant influence on the way they are managed (20).

In our study, APD-A was the most reliable predictor of clinical outcome. This result is consistent with the findings of the meta-analysis published in 2006 by Lee et al. (1). The definition of the APD score chosen for the study was the maximum intrasinusal diameter of the pelvis on transverse sections of the kidney passing through the hilum in the supine position. Indeed, it has been shown that APD can vary according to the patient's hydration status and position (21), but above all according to whether or not the extrarenal pelvis is included (22). A recent study comparing six different techniques for measuring anteroposterior diameter identified significant differences depending on where the measurement was taken (23). In a similar study directly comparing intra- and extra-sinusal diameter on 212 ultrasound scans, a mean diameter difference of 6 ± 6 mm was identified (range 0–22 mm) (24). The ACR multidisciplinary consensus recommends that APD should be measured at the maximum intrasinusal diameter of the renal pelvis.

We set cut-off points at 15 mm antenatally and 20 mm postnatally. These values are based on data from the literature and on commonly studied thresholds (1, 25). We considered an increase of >5 mm between two ultrasound scans to be significant, based on a study showing a possible variation of 3 mm on ultrasound scans repeated every 15 min for 2 h (26).

Twenty-one percent of our patients presented a fUTI. This could seem high though previous reports have found similar rates in children with high grade hydronephrosis (1). We do not perform cystograms in children without ureteral dilation, even after an initial fUTI, if they present significant UPJO. If a child has recurrent fUTI after pyeloplasty, we perform the cystogram. Nevertheless, this did not occur in our series.

It is important to note, that at the level of the individual patient, even APD is of relatively little value. Indeed, in our series, we observe a 40% risk of clinical event in children with APD-A <15 mm vs. an 89% risk for those with APD-A ≥15 mm, but the relative risk (RR) is only 2. This simply means that a child with UPJO and a 3rd trimester APD-A ≥15 mm has twice the risk of being operated on than a child with APD-A <15 mm, which is both significant and vague. This predictive limitation must be taken into account during prenatal counselling.

The strength of our study is that it was carried out on a homogeneous population with standardized prospective follow-up. Our series includes only patients with UPJO, and we choose only to investigate children with significant dilation. Indeed, there are only 2 UTD-A1 patients and only 1 UTD-P1, which is comparable to the literature where most patients diagnosed with UPJO were classified UTD-P2 or -P3 (14). Our inclusion process eliminated UTD-P0 or 1 patients, who are in reality, those whom the score would have identified as not at risk. We could say we did not include children for whom the score would have told us not to investigate.

The purpose of a predictive score should be to predict a clinically significant event that could warrant therapeutic management. This is why we studied significant clinical elements recognized as potential indications for surgical management (27, 28). However, according to our local strategy, only children with increased dilation or a fUTI underwent subsequent surgery.

Finally, the review of each ultrasound scan was centralized and blinded to the children's outcome, which decreases the inherent variability of ultrasound reports.

The limitations of our study are essentially the small number of patients. We cannot rule out for instance the possibility that the predictive capacity of UTD may improve with a larger number of patients. Nevertheless, previous reports have suggested that even on larger populations, when reduced to the specific population of UPJO syndromes, APD remains the criterion of choice and that it should not affect how families are counselled (29, 30). The follow-up is also relatively short, but we believe a prognostic tool needs to identify something that will happen within a limited timeframe to be useful, this is why we chose the limit of 1 year. Interestingly, none of the children who continued simple observation presented a significant event during the second year of life. Furthermore, in our study all clinically significant events occurred within the first year of life and none occurred during the second.

## Conclusion

In our study, the most effective predictive factor of a future significant clinical event on ultrasound was an APD ≥ 15 mm in the 3rd trimester of pregnancy, which predicted a subsequent increase in dilation >5 mm.

## Data Availability

The raw data supporting the conclusions of this article will be made available by the authors, without undue reservation.
